# Supplementary material for: ZapC crosslinks FtsZ filaments through a dual-binding mechanism modulated by the intrinsically disordered linker of FtsZ in Escherichia coli
Source: mBio. 2025 Oct 20;16(11):e02622-25. doi: 10.1128/mbio.02622-25 (PMC12607630; doi:10.1128/mbio.02622-25)
Supplement: Supplemental material — Figures S1-S6; Tables S1-S9. [file mbio.02622-25-s0001.pdf]

Supplementary Information For:

ZapC crosslinks FtsZ filaments through a dual-binding mechanism modulated by the intrinsically disordered linker of FtsZ in *Escherichia coli*

Authors
Ying Li[1,2], Han Gong[1,2], Rui Zhan[1,2], Yuanyuan Cui[1,2], Xiangdong Chen[3], Joe Lutkenhaus[4], and Shishen Du[1, 2]*

Affiliation
1 State Key Laboratory of Metabolism and Regulation in Complex Organisms, College of Life Sciences, Wuhan University, Wuhan, Hubei, China
2 Hubei Key Laboratory of Cell Homeostasis, College of Life Sciences, Wuhan University, Wuhan, Hubei, China
3 State Key Laboratory of Virology and Biosafety, College of Life Sciences, Wuhan University, Wuhan, Hubei, China
4 Department of Microbiology, Molecular Genetics and Immunology, University of Kansas Medical Center, Kansas City, Kansas, USA

*** To whom correspondence should be addressed:**
Shishen Du
State Key Laboratory of Metabolism and Regulation in Complex Organisms,
College of Life Sciences, Wuhan University, Wuhan, Hubei, China
e-mail: ssdu@whu.edu.cn

Supplementary Figure legends

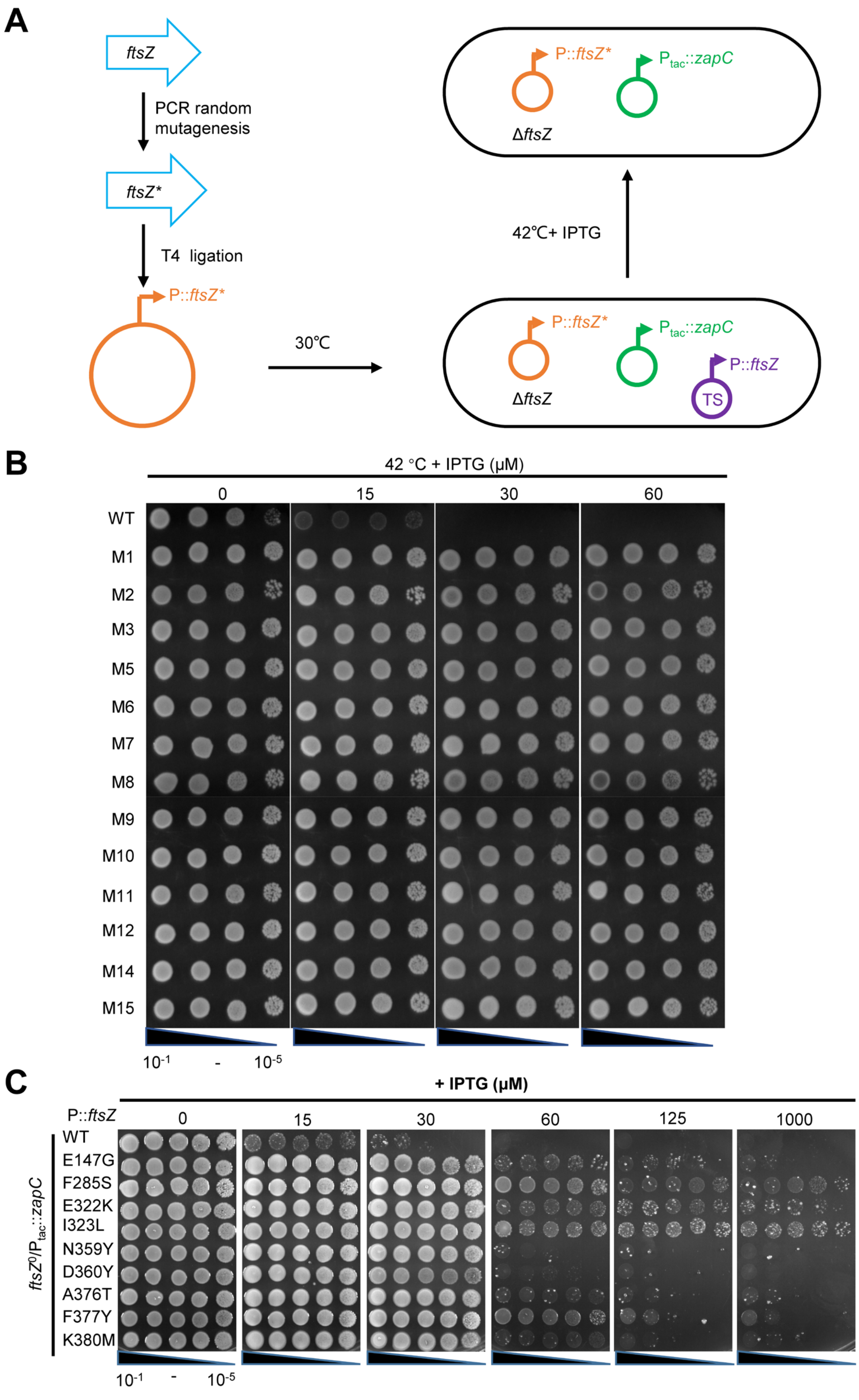

Figure S1. Screening for *ftsZ* mutants resistant to ZapC overexpression. (A) Schematic diagram for screening for FtsZ mutants conferring resistance to ZapC overexpression. *ftsZ* was mutagenized by error-prone PCR, cut and

ligated into the plasmid pBANG112 (p15A, *ftsZ*) to construct an FtsZ mutant library. The library was transformed into strain S17/pKD3C (W3110, *ftsZ*0/pSC101^{ts}, *ftsZ*) harboring plasmid pSD320 (pEXT22, P_{tac}::*zapC*) and transformants selected on LB plates with ampicillin, kanamycin and 60 μM IPTG at 42°C. Transformants that could grow might contain *ftsZ* mutations that were resistant to ZapC overexpression. (B) Spot test to assess the resistance of *ftsZ* mutants to ZapC overexpression. To confirm that the resistance of the transformants to ZapC was due to the harbored plasmid, the plasmids were isolated, re-stransformed into S17/pKD3C containing plasmid pSD320 at 42°C. Transformants were then subjected to spot test to confirm that the resistance to ZapC overexpression was plasmid-linked. (C) A spot test of individual FtsZ mutants displaying resistance to ZapC overexpression toxicity. Plasmid pBANG112 or its derivatives carrying different *ftsZ* alleles were transformed into strain S17/pKD3C harboring plasmid pSD320. Transformants were selected and tested as in (B).

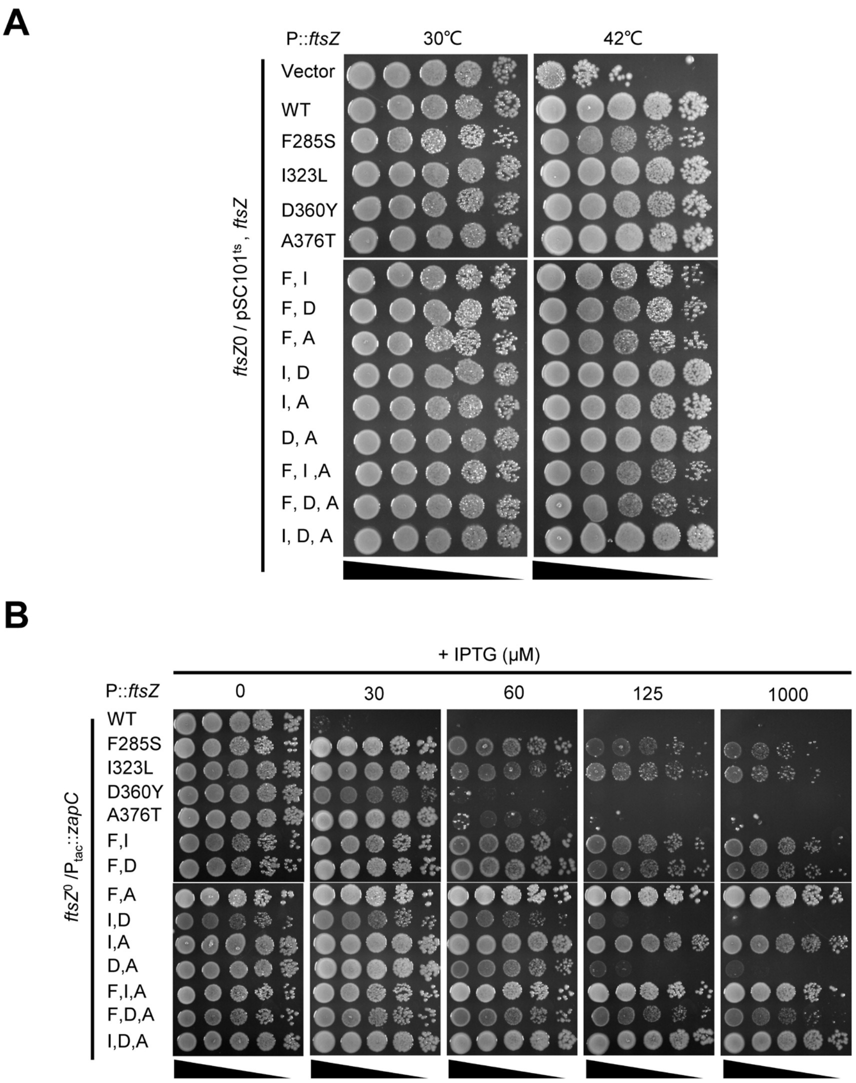

Figure S2. The effect of *ftsZ* mutations on ZapC resistance is additive.
(A) Complementation test of FtsZ mutants. Plasmid pB0, pBANG112 or its derivatives carrying *ftsZ* mutations were transformed into strain S17/pKD3C (W3110, *ftsZ*0/pSC101^{ts}, *ftsZ*) on LB plates with ampicillin at 30°C overnight. The next day, a single transformant of each resulting strain was resuspended in 1 mL of LB medium, serially diluted. 2 μL of each dilution was spot on LB plates with ampicillin. Plates were incubated at 30 and 42°C overnight and photographed. (C) Combination of *ftsZ* mutations provides greater resistance to ZapC overexpression toxicity. The test was performed as in Fig. S1B.

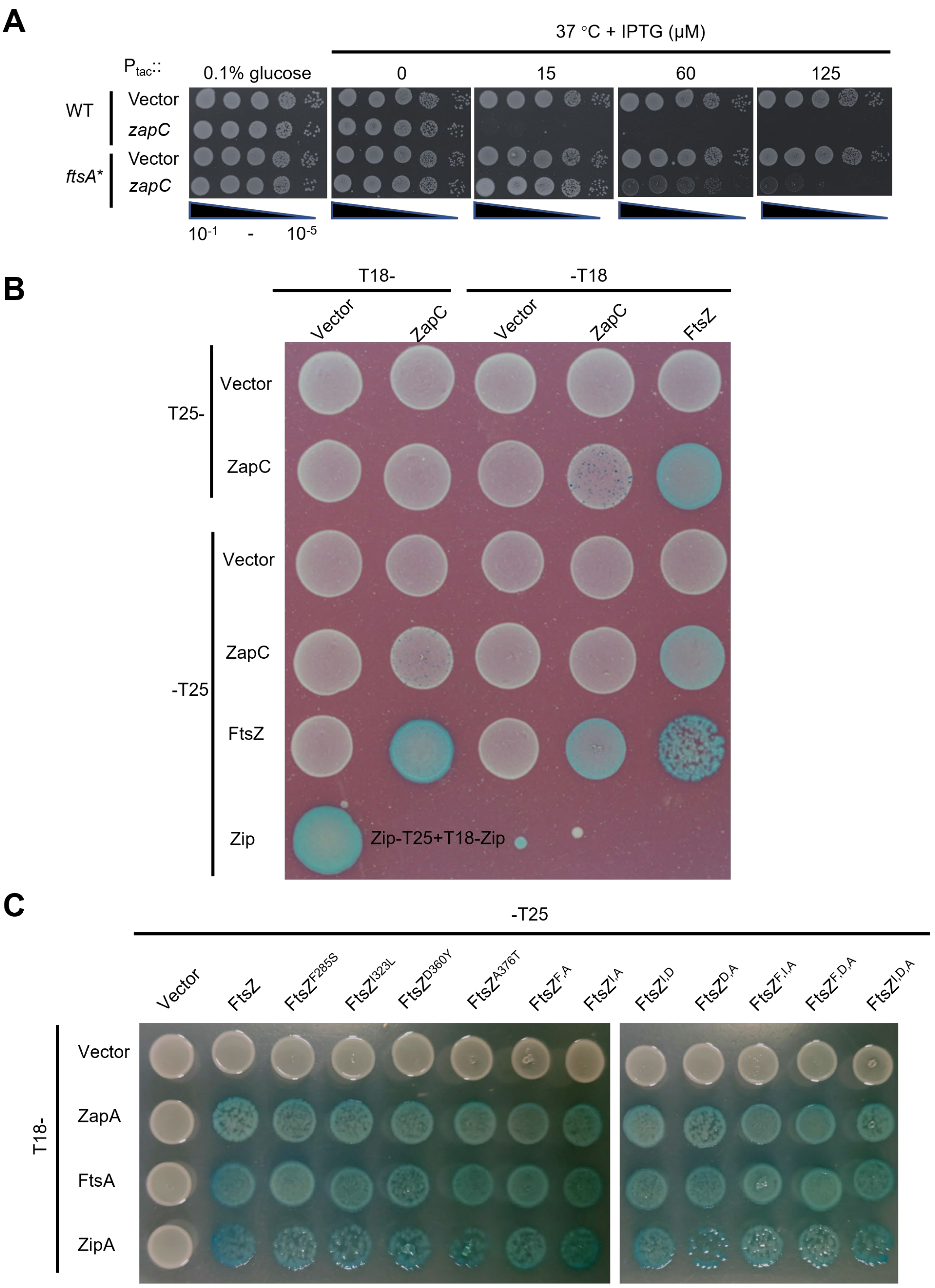

Figure S3. FtsA* (FtsA^{R286W}) provides modest resistance to ZapC overexpression toxicity.

(A) A Spot test to assess the resistance of cells expressing *ftsA** to ZapC overexpression. Plasmid pEXT22 or pSD320 (pEXT22, P_{tac}::*zapC*) was transformed into strain W3110 or PS2343 (W3110, *leu::Tn10 ftsA**) on LB plates with kanamycin at 37°C overnight. 2 μL of each dilution was spot on a LB plate with kanamycin and IPTG. Plates were incubated at 37°C overnight and photographed. (B) Bacterial two hybrid test of the interaction between FtsZ and ZapC. Plasmids pairs were transformed into strain LYA1 (BTH101, *ftsA**), the

next day a single transformant of each resulting strain was resupended in 1 mL LB medium, 2 μL of each culture was spot on LB plates containing antibiotics, 40 μg/mL X-gal and IPTG. Plates were incubated at 30°C for about 24 hours before photographing. (C) Bacterial two hybrid test of the interaction between FtsZ or its mutants and ZapA or other divisome proteins. The test was performed as in (B) except that the strain used was BTH101.

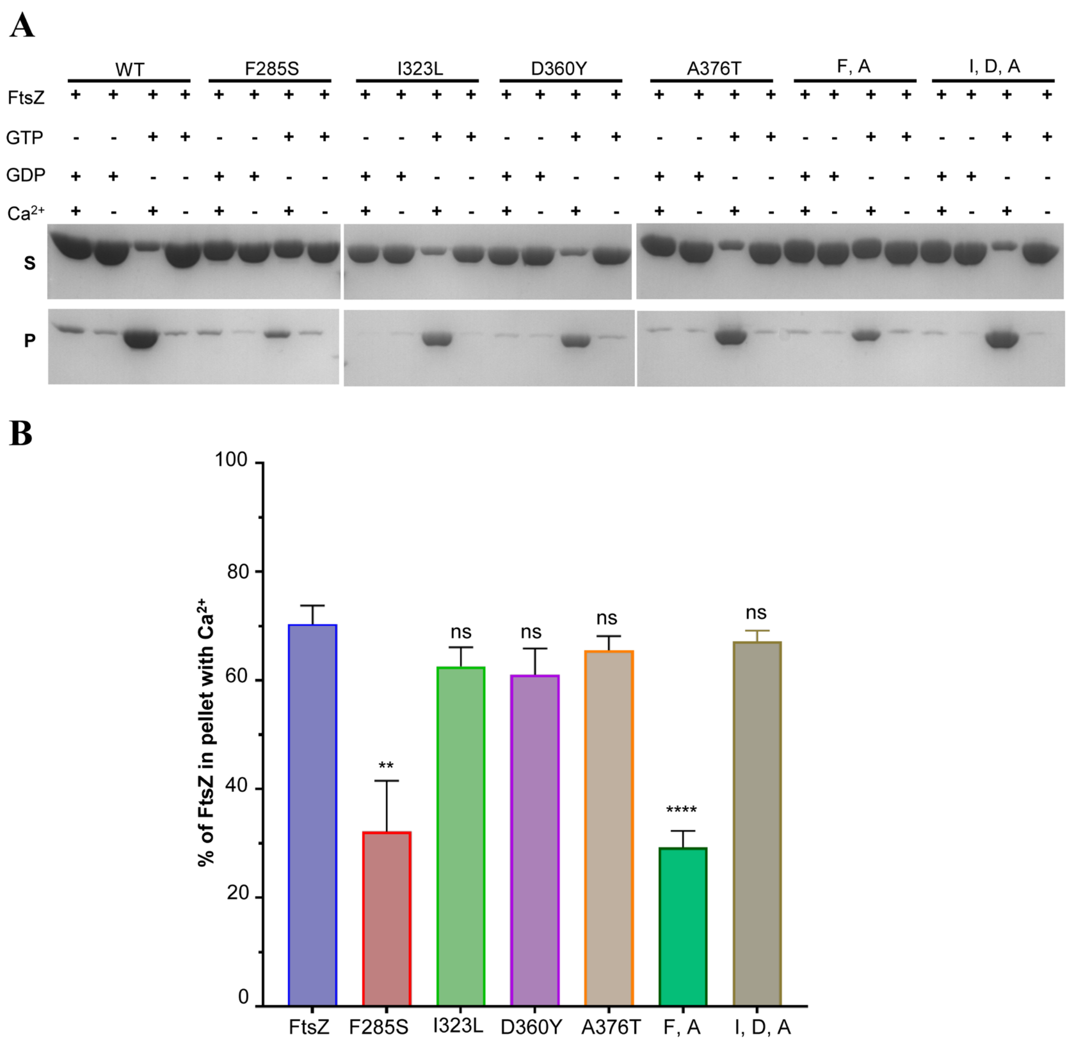

Figure S4. ZapC-resistant FtsZ mutants can polymerize.

(A) Sedimentation assay was carried out to access the ability of FtsZ mutants to polymerize as described in Materials and Methods. FtsZ or its variant (5 μM) was added into polymerization buffer (50 mM HEPES-KOH, 50 mM KCl, 10 mM $MgCl_2$, pH 6.8), GDP/GTP (2.5 mM) and 10 mM Ca^{2+} were added to the reaction in a 50 μL reaction volume. The samples were incubated at room temperature for 5 min before centrifuged, and the pellets and supernatants were analyzed by SDS-PAGE. (B) Quantification of the amount of FtsZ in the pellet in (A). Protein bands were analyzed by Image J and the percentages of FtsZ or its mutants in the pellets verse total amount of protein were plotted. Data were presented as mean values +/- s.d.. Significance of differences is tested relative to wild type FtsZ; **$P < 0.01$, ****$P < 0.0001$; ns, not significant ($P > 0.05$), two-tailed Student's t test.

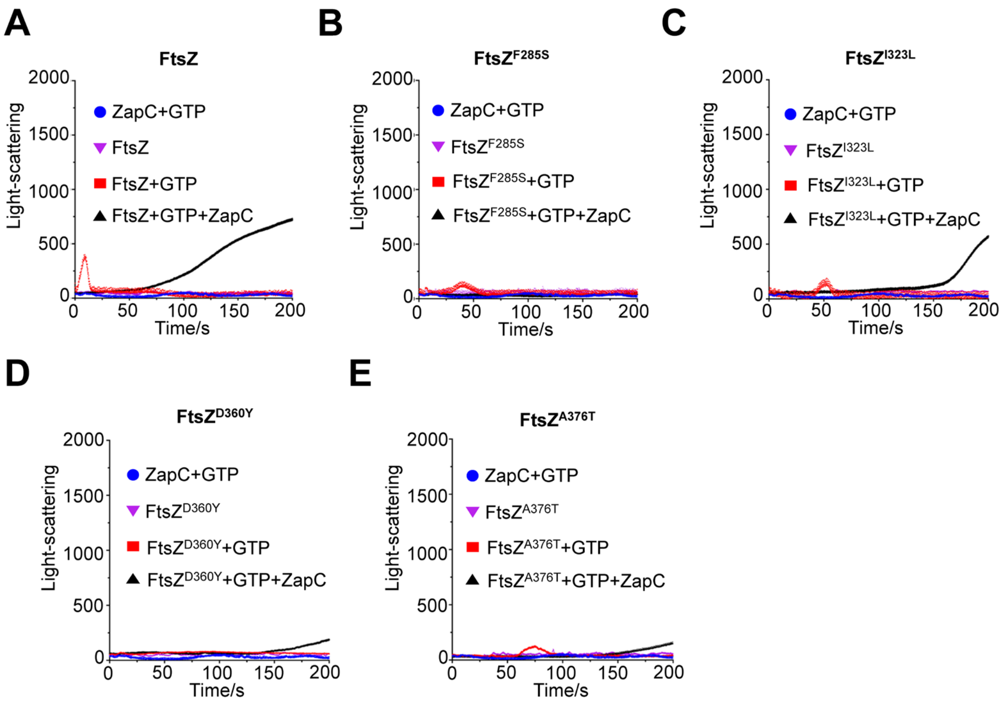

Fig. S5 The influence of ZapC on the assembly of FtsZ or its mutants assayed by 90° light scattering.

Polymerization of FtsZ or its mutants were carried out at room temperature in a cuvette as described in Materials and Methods. Final concentration of FtsZ (A) or its mutants (B-E), and ZapC was kept at 1 μM, GTP was added to a final concentration of 0.5 mM. Each light-scattering measurement was repeated two or three times, with consistent results.

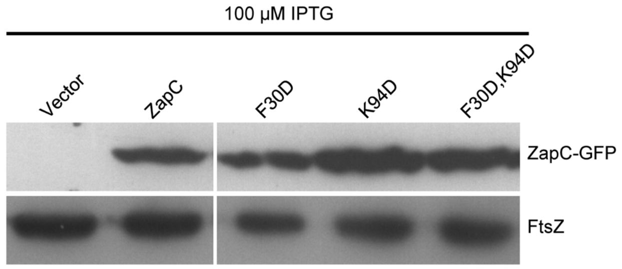

Figure S6. Mutations at the ZapC hydrophobic pocket site do not affect the expression levels of ZapC and FtsZ proteins.
Western blot test of the expression levels of ZapC and its variants. Details about the experimental system and procedures is described in Materials and Methods.

Table S1 Mutations in the ZapC-resistant FtsZ mutants.

Mutant #	Mutations	Resistance to ZapC overexpression (IPTG µM)
M1	I323L, N359Y	> 60
M2	R258H, V260M, A376T	> 60
M3	F285S	> 60
M5	V260M, A376T	> 60
M6	V260A, V292A, F377Y	> 60
M7	E147G, E350G	> 60
M8	E322K, K380M	> 60
M9	I323L, N359Y	> 60
M10	R258H, V260M, A376T	> 60
M11	F285Y	> 60
M12	E250D, N359Y	> 60
M14	Q56L, Q330H, D360Y	> 60
M15	A101G, K380M	> 60

Table S2. Summary of the ZapC-resistant FtsZ substitutions.

Substitution	Resistance to ZapC	Location in the subdomain of FtsZ
E147G	++	Globular
F285S/Y	++++	Globular
E322K	++++	CTL
I323L	++++	CTL
N359Y	+	CTL
D360Y	+	CTL
A376T	++	CTP
F377Y	+++	CTP
K380M	++	CTP

Resistance: “++++”, “+++”, “++”and “+” indicate that the mutation can counteract the toxicity of ZapC induced by 1000、125、60 and 30 μM IPTG.

Table S3. Co-localization of ZapA-mCherry and ZapC-GFP in cells expressing different FtsZ variants.

Strain	Number of ZapC-GFP rings	Number of ZapA-mCherry rings	Co-localization
WT	109	111	98.20%
F285S	13	153	8.50%
I323L	142	152	93.42%
D360Y	105	107	98.10%
A376T	58	133	43.61%
F, I	3	148	2.03%
F, D	3	142	2.11%
F, A	3	164	1.83%
I, D	160	180	88.85%
I, A	26	164	15.85%
D, A	94	240	39.17%
F, I, A	0	145	0.00%
F, I, D	2	178	1.12%
F, D, A	20	158	12.66%

Table S4. Dissociation constants (Kd) of FtsZ variants for ZapC.

Protein	1		2		3			
His-ZapC	KD	R^2	KD	R^2	KD	R^2	X	SD
FtsZ	0.377	0.993	0.395	0.999	0.488	0.990	0.420	0.060
$FtsZ^{F285S}$	5.018	0.996	6.374	0.994	6.134	0.985	5.842	0.724
$FtsZ^{I323L}$	0.579	0.993	0.647	0.995	0.635	0.993	0.620	0.036
$FtsZ^{D360Y}$	0.573	0.993	0.823	0.989	0.735	0.995	0.710	0.127
$FtsZ^{A376T}$	0.859	0.991	0.778	0.994	0.821	0.999	0.819	0.041
$FtsZ^{1-316}$	1.537	0.989	1.909	0.998	1.290	0.994	1.579	0.312
$FtsZ^{1-330}$	1.610	0.952	1.809	0.970	1.590	0.971	1.670	0.121
$FtsZ^{1-370}$	1.538	0.993	1.194	0.993	1.263	0.978	1.332	0.182

Table S5. Dissociation constants (Kd) of ZapC for SUMO-FtsZ or truncated FtsZ variants.

Protein	1		2		3			
ZapC	KD	R^2	KD	R^2	KD	R^2	X	SD
-FtsZ	1.504	0.996	1.279	0.998	1.899	0.999	1.561	0.314
-$FtsZ^{316-383}$	11.21	0.989	10.36	0.992	8.109	0.995	9.893	1.602
-$FtsZ^{370-383}$	11.40	0.996	10.21	0.986	8.061	0.993	9.890	1.692
-$FtsZ^{316-370}$	50.66	0.931	47.22	0.909	77.00	0.909	58.29	16.29
SUMO	Response value < 0.05，no interaction							

Table S6. Dissociation constants (Kd) of FtsZ for His-ZapC or its mutants.

Protein	1		2		3			
FtsZ	KD	R^2	KD	R^2	KD	R^2	X	SD
His-ZapC	0.377	0.993	0.395	0.999	0.364	0.976	0.379	0.016
-ZapC^{F30D}	2.971	0.988	2.551	0.979	3.045	0.975	2.856	0.266
-ZapC^{K94D}	2.142	0.968	1.735	0.970	2.095	0.974	1.991	0.223
-ZapC$^{F, K}$	6.394	0.970	5.160	0.912	4.772	0.966	5.442	0.847

Table S7. Bacterial strains used in this study.

Strain	Genotype	Source /Reference
BTH101	*cya*-99 *araD*139 *galE*15 *galK*16 *rpsL*1 *hsdR*2 *mcrA*1 *mcrB*1	[1]
W3110		Lab collection
LYA1	BTH101, *ftsA*^{R286W}	This study
JS238	MC1061 *malPp::lacIQ srlC::Tn10 recA1*	[2]
LYA4	TB28, *zapA-mCherry cat<>frt*	This study
LYA5/pKD3C	W3110, *leu*::*Tn10* *ftsZ*^{0}/pSC101^{ts}, *ftsZ*; CmR	This study
LYA6/pKD3C	TB28, *zapA-mCherry ftsZ*::*Tn10*/ pSC101^{ts}, *ftsZ*, CmR	This study
S17/pKD3C	W3110, *leu::Tn10* *ftsZ*^{0}/pSC101^{ts}, *ftsZ*; CmR	[3]
S17/pBANG112	W3110, *leu::Tn10 ftsZ*^{0}/ p15A, *ftsZ*; AmR	Lab collection
BL21/pLys	expression strain for pET plasmid	Lab collection
PS2343	W3110, *leu*::*Tn10 ftsA*^{R286W}	[4]

Table S8. Plasmids used in this study.

Plasmid	Genotype	Source / Reference
pEXT22	R100 origin, *kan* P_{tac} promoter	[5]
pSD320	pEXT22, *kan* P_{tac}::*zapC*	This study
pSD346	pDSW209, *bla* P_{206}::*gfp-CcftsZlinker60*	This study
pSD366	pDSW210, *bla* P_{206}::*zapC-l60-gfp*	This study
pKNT25	pSM40, *kan* P_{lac}::-*t25*	[1]
pKT25	pACYC184, *kan* P_{lac}::*t25*-	[1]
pUT18	pMB1, *bla* P_{lac}::-*t18*	[1]
pUT18C	pMT18C, *bla* P_{lac}::*t18*-	[1]
pKD46	pSC101^{ts}, *bla* P_{ara}::*gam-bet-exo*	[6]
pSD323	pQE80, *bla* P_{lac}::6XHis-*zapC*	This study
pBANG112	p15A, *bla ftsZ*	[3]
pB0	p15A, *bla ftsZ* frameshift mutation	This study
pUT18C-zapA	pMT18C, *bla* P_{lac}::*t18-zapA*	This study
pUT18C-ftsA	pMT18C, *bla* P_{lac}::*t18-ftsA*	This study
pUT18C-zipA	pMT18C, *bla* P_{lac}::*t18-zipA*	This study
pUT18C-slmA	pMT18C, *bla* P_{lac}::*t18-slmA*	This study
pUT18C-minC	pMT18C, *bla* P_{lac}::*t18-minC*	This study
pLY44	pDSW210, *kan* P_{206}::*zapC-l60-gfp*	This study
pLY225	p15A, *bla ftsZ*^{E147G}	This study
pB1	p15A, *bla ftsZ*^{F285S}	This study
pLY226	p15A, *bla ftsZ*^{E322K}	This study
pB2	p15A, *bla ftsZ*^{I323L}	This study
pB3	p15A, *bla ftsZ*^{N359Y}	This study
pLY227	p15A, *bla ftsZ*^{D360Y}	This study
pB4	p15A, *bla ftsZ*^{A376T}	This study
pLY228	p15A, *bla ftsZ*^{F377Y}	This study
pLY229	p15A, *bla ftsZ*^{K380M}	This study
pLY331	p15A, *bla ftsZ*$^{F285S, I323L}$	This study
pLY332	p15A, *bla ftsZ*$^{F285S, D360Y}$	This study
pB5	p15A, *bla ftsZ*$^{F285S, A376T}$	This study
pLY242	p15A, *bla ftsZ*$^{I323L, D360Y}$	This study
pB6	p15A, *bla ftsZ*$^{I323L, A376T}$	This study
pLY333	p15A, *bla ftsZ*$^{D360Y, A376T}$	This study
pLY334	p15A, *bla ftsZ*$^{F285S, I323L, D360Y}$	This study
pB7	p15A, *bla ftsZ*$^{F285S, I323L, A376T}$	This study
pB8	p15A, *bla ftsZ*$^{F285S, D360Y, A376T}$	This study
pLY333	p15A, *bla ftsZ*$^{D360Y, A376T}$	This study
pLY334	p15A, *bla ftsZ*$^{F285S, I323L, D360Y}$	This study
pB7	p15A, *bla ftsZ*$^{F285S, I323L, A376T}$	This study
pB8	p15A, *bla ftsZ*$^{F285S, D360Y, A376T}$	This study
pLY247	p15A, *bla ftsZ*$^{I323L, D360Y, A376T}$	This study

pLY31	p15A, *bla ftsZ*^{E237A}	This study
pLY32	p15A, *bla ftsZ*^{E241A}	This study
pLY33	p15A, *bla ftsZ*^{S245A}	This study
pLY34	p15A, *bla ftsZ*^{E250A}	This study
pLY35	p15A, *bla ftsZ*^{D253A}	This study
pLY36	p15A, *bla ftsZ*^{T281A}	This study
pLY143	p15A, *bla ftsZ*^{F285Y}	This study
pLY144	p15A, *bla ftsZ*^{F285A}	This study
pLY145	p15A, *bla ftsZ*^{F285E}	This study
pLY146	p15A, *bla ftsZ*^{F285L}	This study
pLY149	p15A, *bla ftsZ*^{F285K}	This study
pLY193	pEXT22, *kan* P$_{tac}$::*zapC*^{F30D}	This study
pLY185	pEXT22, *kan* P$_{tac}$::*zapC*^{K94D}	This study
pLY319	pEXT22, *kan* P$_{tac}$::*zapC*$^{F30D, K94D}$	This study
pLY363	pEXT22, *kan* P$_{tac}$::*zapC*^{E16A}(GAG-GCG)	This study
pLY364	pEXT22, *kan* P$_{tac}$::*zapC*^{E16K}(GAG-AAG）	This study
pLY365	pEXT22, *kan* P$_{tac}$::*zapC*^{H17A} （CAC-GCC)	This study
pLY366	pEXT22, *kan* P$_{tac}$::*zapC*^{H17D}(CAC-GAC）	This study
pLY367	pEXT22, *kan* P$_{tac}$::*zapC*^{L29A}(CTA-GCA）	This study
pLY368	pEXT22, *kan* P$_{tac}$::*zapC*^{L29D}(CTA-GAC)	This study
pLY211	pDSW210, *kan* P$_{206}$::*zapC*^{F30D}*-l60-gfp*	This study
pLY216	pDSW210, *kan* P$_{206}$::*zapC*^{K94D}*-l60-gfp*	This study
pLY321	pDSW210, *kan* P$_{206}$::*zapC*$^{F30D, K94D}$*-l60-gfp*	This study
pZT18	pMB1, *bla* P$_{lac}$::*ftsZ-t18*	This study
pZT25	pACYC184, *kan* P$_{lac}$::*ftsZ-t25*	This study
pLY1	pMT18C, *bla* P$_{lac}$::*t18-zapC*	This study
pLY2	pMB1, *bla* P$_{lac}$::*zapC-t18*	This study
pLY3	pACYC184, *kan* P$_{lac}$::*t25-zapC*	This study
pLY4	pSM40, *kan* P$_{lac}$::*zapC-t25*	This study
pLY5	pACYC184, *kan* P$_{lac}$::*ftsZ*^{F285S}*-t25*	This study
pLY6	pACYC184, *kan* P$_{lac}$::*ftsZ*^{I323L}*-t25*	This study
pLY259	pACYC184, *kan* P$_{lac}$::*ftsZ*^{D360Y}*-t25*	This study
pLY8	pACYC184, *kan* P$_{lac}$::*ftsZ*^{A376T}*-t25*	This study
pLY335	pACYC184, *kan* P$_{lac}$::*ftsZ*$^{F285S, I323L}$*-t25*	This study
pLY336	pACYC184, *kan* P$_{lac}$::*ftsZ*$^{F285S, D360Y}$*-t25*	This study
pLY9	pACYC184, *kan* P$_{lac}$::*ftsZ*$^{F285S, A376T}$*-t25*	This study
pLY262	pACYC184, *kan* P$_{lac}$::*ftsZ*$^{I323L, D360Y}$*-t25*	This study
pLY11	pACYC184, *kan* P$_{lac}$::*ftsZ*$^{I323L, A376T}$*-t25*	This study
pLY337	pACYC184, *kan* P$_{lac}$::*ftsZ*$^{D360Y, A376T}$*-t25*	This study
pLY13	pACYC184, *kan* P$_{lac}$::*ftsZ*$^{F285S, I323L, A376T}$*-t25*	This study
pB9	pACYC184, *kan* P$_{lac}$::*ftsZ*$^{F285S, D360Y, A376T}$*-t25*	This study
pB10	pACYC184, *kan* P$_{lac}$::*ftsZ*$^{I323L, D360Y, A376T}$*-t25*	This study
pLY272	pMT18C, *bla* P$_{lac}$::*t18-zapC*^{F30D}	This study
pLY16	pMT18C, *bla* P$_{lac}$::*t18-zapC*^{K94D}	This study

pLY315	pMT18C, *bla* P_{lac}::*t18-zapC*$^{F30D, K94D}$	This study
pLY17	pE-SMMO-Amp, *bla* P_{T7}::6X*his-sumo-ftsZ*	This study
pLY19	pE-SMMO-Amp, *bla* P_{T7}::6X*his-sumo-ftsZ*^{F285S}	This study
pLY20	pE-SMMO-Amp, *bla* P_{T7}::6X*his-sumo-ftsZ*^{I323L}	This study
pLY339	pE-SMMO-Amp, *bla* P_{T7}::6X*his-sumo-ftsZ*^{D360Y}	This study
pLY22	pE-SMMO-Amp, *bla* P_{T7}::6X*his-sumo-ftsZ*^{A376T}	This study
pLY343	pE-SMMO-Amp, *bla* P_{T7}::6X*his-sumo-ftsZ*$^{1-316}$	This study
pLY340	pE-SMMO-Amp, *bla* P_{T7}::6X*his-sumo-ftsZ*$^{1-330}$	This study
pLY341	pE-SMMO-Amp, *bla* P_{T7}::6X*his-sumo-ftsZ*$^{1-370}$	This study
pLY342	pE-SMMO-Amp, *bla* P_{T7}::6X*his-sumo-ftsZ*$^{316-383}$	This study
pLY28	pE-SMMO-Amp, *bla* P_{T7}::6X*his-sumo-zapC*	This study
pLY950	pQE80L, *bla* P_{lac}::6X*his-ftsZ*$^{1-316}$	This study
pLY323	pQE80L, *bla* P_{lac}::6X*his-zapC*^{F30D}	This study
pLY23	pQE80L, *bla* P_{lac}::6X*his-zapC*^{K94D}	This study
pLY317	pQE80L, *bla* P_{lac}::6X*his-zapC*$^{F30D, K94D}$	This study

Table S9. Primers used in this study.

Primer name	Sequence
FtsZ-F	CAGTGAGCTCTTAGTTACTTAGG
FtsZ-R	ATCGGCCGGGAAATCTACCG
FtsZ-E147G-F	CGTATGGCATTCGCGGGGCAGGGGATCACTGAAC
FtsZ-E147G-R	GTTCAGTGATCCCCTGCCCCGCGAATGCCATACG
FtsZ-F285S-F	GTAACACCATCCGTGCATCTGCTTCCGACAACGCG
FtsZ-F285S-R	CGCGTTGTCGGAAGCAGATGCACGGATGGTGTTAC
FtsZ-E322K-F	CATGGACAAACGTCCTAAAATCACTCTGGTGACC
FtsZ-E322K-R	GGTCACCAGAGTGATTTTAGGACGTTTGTCCATG
FtsZ-I323L-F	GGACAAACGTCCTGAACTCACTCTGGTGACCAATAAG
FtsZ-I323L-R	TATTGGTCACCAGAGTGAGTTCAGGACGTTTGTCCAT
FtsZ-N359Y-F	CCGGTTGCTAAAGTCGTGTATGACAATGCGCCGCAAAC
FtsZ-N359Y-R	GTTTGCGGCGCATTGTCATACACGACTTTAGCAACCGG
FtsZ-D360Y-F	GCTAAAGTCGTGAATTACAATGCGCCGCAAACTG
FtsZ-D360Y-R	CAGTTTGCGGCGCATTGTAATTCACGACTTTAGC
FtsZ-A376T-F	GATTATCTGGATATCCCAACATTCCTGCGTAAGCAAG
FtsZ-A376T-R	CTTGCTTACGCAGGAATGTTGGGATATCCAGATAATC
FtsZ-F377Y-F	CTGGATATCCCAGCATACCTGCGTAAGCAAGCTG
FtsZ-F377Y-R	CAGCTTGCTTACGCAGGTATGCTGGGATATCCAG
FtsZ-K380M-F	CCAGCATTCCTGCGTATGCAAGCTGATTAAGAA
FtsZ-K380M-R	TTCTTAATCAGCTTGCATACGCAGGAATGCTGG
FtsZ-E322K,I323L-F	CATGGACAAACGTCCTAAACTCACTCTGGTGACC
FtsZ-E322K,I323L-R	GGTCACCAGAGTGAGTTTAGGACGTTTGTCCATG
FtsZ-A376T,F377Y-F	GATTATCTGGATATCCCAACATACCTGCGTAAGCAAG
FtsZ-A376T,F377Y-R	CTTGCTTACGCAGGTATGTTGGGATATCCAGATAATC
FtsZ-A376T,F377Y, K380M-F	CTGGATATCCCAACATACCTGCGTATGCAAGCTG
FtsZ-A376T,F377Y, K380M-R	CAGCTTGCATACGCAGGTATGTTGGGATATCCAG
FtsZ-F285Y-F	GTAACACCATCCGTGCATATGCTTCCGACAACGCG
FtsZ-F285Y-R	CGCGTTGTCGGAAGCATATGCACGGATGGTGTTAC
FtsZ-F285A-F	GTAACACCATCCGTGCAGCTGCTTCCGACAACGCG
FtsZ-F285A-R	CGCGTTGTCGGAAGCAGCTGCACGGATGGTGTTAC
FtsZ-F285E-F	GTAACACCATCCGTGCAGAAGCTTCCGACAACGCG
FtsZ-F285E-R	CGCGTTGTCGGAAGCTTCTGCACGGATGGTGTTAC
FtsZ-F285L-F	GTAACACCATCCGTGCACTTGCTTCCGACAACGCG
FtsZ-F285L-R	CGCGTTGTCGGAAGCAAGTGCACGGATGGTGTTAC
FtsZ-F285K-F	GTAACACCATCCGTGCAAAAGCTTCCGACAACGCG
FtsZ-F285K-R	CGCGTTGTCGGAAGCTTTTGCACGGATGGTGTTAC
pZT25-F	CCCAAGCTTGTAGGCGACAGGCACAAATCGGAG
pZT25-R	GCGGGATCCTCATCAGCTTGCTTACGCAGGA
pLY1-F	CGCGGATCCACGAATTAAACCAGACGATAA
pLY1-R	CGGGGTACCTTAGACTGCCTGTTCGAGGCT

pLY2-F	CGCGGATCCGTAAAATAGGGTAGGGGAGAGGCA
pLY2-R	CGGGGTACCCGGACTGCCTGTTCGAGGCTGA
pLY3-F	CGCGGATCCACGAATTAAACCAGACGATAA
pLY3-R	CGGGGTACCTTAGACTGCCTGTTCGAGGCT
pLY4-F	CGCGGATCCGTAAAATAGGGTAGGGGAGAGGCA
pLY4-R	CGGGGTACCCGGACTGCCTGTTCGAGGCTGA
BsaI-out-F	CGCGGTCTCAAGGTTTTGAACCAATGGAACTT
316XbaI-out-	CGCGGTCTCAGGCATGGACAAACGTCCTGAAA
316XbaI-out-R	CGCTCTAGATTAGATACCTGTCGCAACAAC
330XbaI-out-R	CGCTCTAGATTACTGCTTATTGGTCACCAG
370XbaI-out-R	CGCTCTAGATTAATCCGGCTCTTTCGCAGT
XbaI-out-R	CGCTCTAGATTAATCAGCTTGCTTACG
FtsZ-E237A-F	GGTGAAGACCGTGCGGCAGAAGCTGCTGAAATG
FtsZ-E237A-R	CATTTCAGCAGCTTCTGCCGCACGGTCTTCACC
FtsZ-E241A-F	GCGGAAGAAGCTGCTGCAATGGCTATCTCTTCTC
FtsZ-E241A-R	GAGAAGAGATAGCCATTGCAGCAGCTTCTTCCGC
FtsZ-S245A-F	GCTGAAATGGCTATCGCTTCTCCGCTGCTGGAAG
FtsZ-S245A-R	CTTCCAGCAGCGGAGAAGCGATAGCCATTTCAGC
FtsZ-E249A-F	ATCTCTTCTCCGCTGGCGGAAGATATCGACCTG
FtsZ-E249A-R	CAGGTCGATATCTTCCGCCAGCGGAGAAGAGAT
FtsZ-I252A-F	CCGCTGCTGGAAGATGCCGACCTGTCTGGCGCG
FtsZ-I252A-R	CGCGCCAGACAGGTCGCGATCTTCCAGCAGCGG
FtsZ-D253A-F	CTGCTGGAAGATATCGCCCTGTCTGGCGCGCGC
FtsZ-D253A-R	GCGCGCGCCAGACAGGGCGATATCTTCCAGCAG
FtsZ-T281A-F	GAAACGGTAGGTAACGCCATCCGTGCATTTGCT
FtsZ-T281A-R	AGCAAATGCACGGATGGCGTTACCTACCGTTTC
zapC-W9D-F	AAACCAGACGATAACGACCGTTGGTATTACGATG
zapC-W9D-R	CATCGTAATACCAACGGTCGTTATCGTCTGGTTT
zapC-F30D-F	GCCAATGGTATGCTAGACCGCTCACGTTTTGCG
zapC-F30D-R	CGCAAAACGTGAGCGGTCTAGCATACCATTGGC
zapC-K94D-F	AAACCGCAAATGCCGGACAGCTGGCATTTTGTTTC
zapC-K94D-R	GAAACAAAATGCCAGCTGTCCGGCATTTGCGGTTT
pSD323-out-F	TGCCACCTGACGTCTAAGAA
pSD323-out-R	CAACGGTGGTATATCCAGTG
SUMO-out-F	CCAGAAGTCAAGCCTGAGAC
SUMO-out-R	TGGCAAGTGTAGCGGTCACG
ftsZ-out-F	GGAGCCGTATTATTCGACGG
ftsZ-out-R	CGTCAGGGTGACTTTCTTGC
pLY4-out-F	ACTCATTAGGCACCCCAGGC
pLY4-out-R	TGTCTGTAAGCGGATGCCGG
pKT25-out-F	CGGTGACCGATTACCTGGCG
pKT25-out-R	CCATTCGCCATTCAGGCTGC
pZT18-out-R	CGCGAGCGATTTTCCACAAC
pZT25-out-R	TGTCTGTAAGCGGATGCCGG

HindIII-kan-F	GCAAGCTTCTGTTTTGGCGGATGAGAG
ScaI-kan-R	GCAGTACTTCAGAAGAACTCGTCAAGAA
SUMO-ZapC-F	CGGGTCTCAAGGTCGAATTAAACCAGACGAT
SUMO-ZapC-R	CGTCTAGATTAGACTGCCTGTTCGAG
pLY950-F	ATCGCATCACCATCACCATCACTTTGAACCAATGGAACTTACCA
pLY950-R	TCCAAGCTCAGCTAATTAAGCTTTAGCCGATACCTGTCGCAACA
T18-slmA-F	CTGCAGGTCGACTCTAGAGGATCCGGCAGAAAAACAAACTGCGAA
T18-slmA-R	ATTACTTAGTTATATCGATGAATTCTTACTGCAACTGTGCCGCAA
T18-minC-F	CTGCAGGTCGACTCTAGAGGATCCGTCAAACACGCCAATCGAGCT
T18-minC-R	ATTACTTAGTTATATCGATGAATTCTCAATTTAACGGTTGAACGG

Construction of strains and plasmids

LYA1

The strain LYA1 (BTH101, *leu::Tn10 ftsA*^{R286W}) was constructed by introducing *leu::Tn10 ftsA*^{R286W} from PS2343 (W3110, *leu::Tn10 ftsA*^{R286W}) into BTH101 through P1 transduction.

LYA5/pKD3C

The strain LYA5/pKD3C (W3110, *leu*::*Tn10 ftsZ*^{0}/pSC101^{ts}, *ftsZ*; CmR) was constructed by P1 transduction of the *leu*::*Tn10* cassette from S3 (W3110, *leu*::*Tn10*) into strain S17/pKD3C (W3110, *ftsZ*^{0} /pSC101^{ts}, *ftsZ*; CmR). Transductants were selected on LB plates with 7.5 μg/mL chloramphenicol, 12.5 μg/mL tetracycline and 1mM sodium citrate at 30 and 42 °C. Transductants only grown at 30 °C with 7.5 μg/mL chloramphenicol, 12.5 μg/mL tetracycline.

LYA6/pKD3C

The strain LYA6/pKD3C (TB28, *zapA*-*mCherry leu*::*Tn10 ftsZ*^{0}/pSC101^{ts}, *ftsZ*; CmR) was constructed by P1 transduction of the *leu*::*Tn10 ftsZ*^{0} cassette from LYA5/pKD3C (W3110, *leu*::*Tn10 ftsZ*^{0}/pSC101^{ts}, *ftsZ*; CmR) into strain LYA4/pKD3C (TB28, *zapA-mCherry cat*<>*frt* /pSC101^{ts}, *ftsZ*; CmR). Transductants were selected on LB plates with 7.5 μg/mL chloramphenicol, 12.5 μg/mL tetracycline and 1mM sodium citrate at 30 and 42°C. Transductants only grown at 30 °C with 7.5 μg/mL chloramphenicol, 12.5 μg/mL tetracycline.

pZT18

The plasmid pZT18 (pUT18, P_{lac}::*ftsZ-t18*) was constructed by ligation of an BamHI/HindIII digested DNA fragment containing *ftsZ* into pUT18 digested with the same enzymes. The DNA fragment was amplified from plasmid pBANG112 (p15A, *ftsZ bla*) using primers pZT18-F/R.

pZT25

The plasmid pZT18 (pKNT25, P_{lac}::*ftsZ-t25*) was constructed by ligation of an

BamHI/HindIII digested DNA fragment containing *ftsZ* into pKNT25 digested with the same enzymes. The DNA fragment was amplified from plasmid pBANG112 (p15A, *ftsZ bla*) using primers pZT25-F/R.

pZT25 derivatives

Derivatives of pZT25 (pKNT25, P_{lac}::*ftsZ-t25*) harboring different alleles of *ftsZ* were created by site-directed mutagenesis using the primer pairs listed in Table S9.

pSD320 derivatives

Derivatives of pSD320 (pEXT22, P_{tac}::*zapC kan*) harboring different alleles of *zapC* were created by site-directed mutagenesis using the primer pairs listed in Table S9.

pSD323 derivatives

Derivatives of pSD323 (pQE80, *bla* P_{lac}::6X*his-zapC*) harboring different alleles of *zapC* were created by site-directed mutagenesis using the primer pairs listed in Table S9.

pSD366 derivatives

Derivatives of pSD366 (pDSW210, *bla* P_{206}::*zapC-l60-gfp*) harboring different alleles of *zapC* were created by site-directed mutagenesis using the primer pairs listed in Table S9.

pBANG112 derivatives

Derivatives of pBANG112 (p15A, *bla ftsZ*) harboring different alleles of *ftsZ* were created by site-directed mutagenesis using the primer pairs listed in Table S9.

pLY1

The plasmid pLY1 (pUT18C, P_{lac}::*t18-zapC*) was constructed by ligation of an BamHI/KpnI digested DNA fragment containing *zapC* into pUT18C digested with the same enzymes. The DNA fragment was amplified from plasmid pSD320 (pEXT22, *kan* P_{tac}::*zapC*) using primers pLY3-F/R.

pLY1 derivatives

Derivatives of pLY1 (pUT18C, P_{lac}::*t18-zapC*) harboring different alleles of *zapC* were created by site-directed mutagenesis using the primer pairs listed in Table S9.

pLY2

The plasmid pLY2 (pUT18, P_{lac}::*zapC-t18*) was constructed by ligation of an BamHI/KpnI digested DNA fragment containing *zapC* into pUT18 digested with the same enzymes. The DNA fragment was amplified from plasmid pSD320 (pEXT22, *kan* P_{tac}::*zapC*) using primers pLY4-F/R.

pLY3

The plasmid pLY3 (pKT25, P_{lac}::*t25-zapC*) was constructed by ligation of an

BamHI/KpnI digested DNA fragment containing *zapC* into pKT25 digested with the same enzymes. The DNA fragment was amplified from plasmid pSD320 (pEXT22, *kan* P_{tac}::*zapC*) using primers pLY3-F/R.

pLY4
The plasmid pLY4 (pKNT25, P_{lac}::*zapC-t25*) was constructed by ligation of an BamHI/KpnI digested DNA fragment containing *zapC* into pKNT25 digested with the same enzymes. The DNA fragment was amplified from plasmid pSD320 (pEXT22, *kan* P_{tac}::*zapC*) using primers pLY4-F/R.

pLY17
The plasmid pLY17 (pE-SUMO-Amp, *bla* P_{T7}::6×*his-sumo-ftsZ*) was constructed by ligation of an XbaI/BsaI digested DNA fragment containing *ftsZ* into pE-SUMO-Amp digested with the BsaI enzymes. The DNA fragment was amplified from plasmid pBANG112 (p15A, *bla ftsZ*) using primers BsaI-out-F and XbaI-out-R.

pLY17 derivatives
Derivatives of pLY17 (pE-SUMO-Amp, *bla* P_{T7}::6×*his-sumo-ftsZ*) harboring different alleles of *ftsZ* were created by site-directed mutagenesis using the primer pairs listed in S3 table.

pLY340
The plasmid pLY340 (pE-SUMO-Amp, *bla* P_{T7}::6×*his-sumo-ftsZ*$^{1\text{-}330}$) was constructed by ligation of an XbaI/BsaI digested DNA fragment containing *ftsZ*$^{1\text{-}330}$ into pE-SUMO-Amp digested with the BsaI enzymes. The DNA fragment was amplified from plasmid pBANG112 (p15A, *bla ftsZ*) using primers BsaI-out-F and 330XbaI-out-R.

pLY341
The plasmid pLY341 (pE-SUMO-Amp, *bla* P_{T7}::6×*his-sumo-ftsZ*$^{1\text{-}370}$) was constructed by ligation of an XbaI/BsaI digested DNA fragment containing *ftsZ*$^{1\text{-}370}$ into pE-SUMO-Amp digested with the BsaI enzymes. The DNA fragment was amplified from plasmid pBANG112 (p15A, *bla ftsZ*) using primers BsaI-out-F and 370XbaI-out-R.

pLY342
The plasmid pLY342 (pE-SUMO-Amp, *bla* P_{T7}::6×*his-sumo-ftsZ*$^{316\text{-}383}$) was constructed by ligation of an XbaI/BsaI digested DNA fragment containing *ftsZ*$^{316\text{-}383}$ into pE-SUMO-Amp digested with the BsaI enzymes. The DNA fragment was amplified from plasmid pBANG112 (p15A, *bla ftsZ*) using primers 316BsaI-out-F and XbaI-out-R.

pLY343
The plasmid pLY343 (pE-SUMO-Amp, *bla* P_{T7}::6×*his-sumo-ftsZ*$^{1\text{-}316}$) was constructed by ligation of an XbaI/BsaI digested DNA fragment containing *ftsZ*$^{1\text{-}316}$ into pE-SUMO-Amp digested with the BsaI enzymes. The DNA fragment was amplified from plasmid pBANG112 (p15A, *bla ftsZ*) using primers BsaI-out-F and 316XbaI-out-R.

pLY28

The plasmid pLY28 (pE-SUMO-Amp, *bla* P_{T7}::6×*his-sumo-zapC*) was constructed by ligation of an XbaI/BsaI digested DNA fragment containing *zapC* into pE-SUMO-Amp digested with the BsaI enzymes. The DNA fragment was amplified from plasmid pSD320 (pEXT22, *kan* P_{tac}::*zapC*) using primers SUMO-ZapC-F/R.

pLY44

The plasmid pLY44 (pDSW210, *kan* P_{206}::*zapC*-l60-*gfp*) was constructed by ligation of an ScaI/HindIII digested DNA fragment containing *kan* into pSD366 (pDSW210, *bla* P_{206}::*zapC-l60-gfp*) digested with the same enzymes. The DNA fragment was amplified from plasmid pEXT22 using primers HindIII-kan-F and ScaI-kan-R.

pLY44 derivatives

Derivatives of pLY44 (pDSW210, *kan* P_{206}::*zapC-l60-gfp*) harboring different alleles of *zapC* were created by site-directed mutagenesis using the primer pairs listed in Table S9.

pLY950

The plasmid pLY950 (pSD323, P_{lac}::6×*his*-*ftsZ*) was constructed by ligation of a BamHI/HindIII digested DNA fragment containing *ftsZ* into pSD323 digested with the same enzymes. The DNA fragment was amplified from plasmid pBANG112 (p15A, *bla ftsZ*) using primers pLY950-F/R.

References:

1. Karimova, G., Pidoux, J., Ullmann, A., and Ladant, D. (1998). A bacterial two-hybrid system based on a reconstituted signal transduction pathway. Proc Natl Acad Sci U S A *95*, 5752-5756. 10.1073/pnas.95.10.5752.
2. Pichoff, S., and Lutkenhaus, J. (2005). Tethering the Z ring to the membrane through a conserved membrane targeting sequence in FtsA. Mol Microbiol *55*, 1722-1734. 10.1111/j.1365-2958.2005.04522.x.
3. Shen, B., and Lutkenhaus, J. (2009). The conserved C-terminal tail of FtsZ is required for the septal localization and division inhibitory activity of MinC(C)/MinD. Mol Microbiol *72*, 410-424. 10.1111/j.1365-2958.2009.06651.x.
4. Pichoff, S., Shen, B., Sullivan, B., and Lutkenhaus, J. (2012). FtsA mutants impaired for self-interaction bypass ZipA suggesting a model in which FtsA's self-interaction competes with its ability to recruit downstream division proteins. Mol Microbiol *83*, 151-167. 10.1111/j.1365-2958.2011.07923.x.
5. Dykxhoorn, D.M., St Pierre, R., and Linn, T. (1996). A set of compatible tac promoter expression vectors. Gene *177*, 133-136. 10.1016/0378-1119(96)00289-2.
6. Datsenko, K.A., and Wanner, B.L. (2000). One-step inactivation of chromosomal genes in *Escherichia coli* K-12 using PCR products. Proc Natl Acad Sci U S A *97*, 6640-6645.

10.1073/pnas.120163297.
